# Generational effect on nurses’ work values, engagement, and satisfaction in an acute hospital

**DOI:** 10.1186/s12912-023-01256-2

**Published:** 2023-03-30

**Authors:** Si Hui Evangeline Tan, Guey Fong Chin

**Affiliations:** Khoo Teck Puat Hospital, 90 Yishun Central, 768828 Yishun, Singapore

**Keywords:** Generational effect, Generational differences, Values, Engagement, Satisfaction, Baby Boomers, Generation X, Generation Y, Millennials, Generation Z

## Abstract

**Background:**

The present nursing workforce comprises four generational of nurses working side–by–side. While such a generation blend adds invaluable diversity to the workforce, it also brings added complexity. The study aimed to describe and summarise work values and attitudes of four nursing generations, namely Baby boomers, Generation X, Y and Z.

**Method:**

A cross-sectional questionnaire study was adopted. A total of 778 nurses from an acute hospital in Singapore completed the online questionnaire. The Work Value and Attitude scale measuring seven constructs (Work Centrality, Non-compliance, Technology Challenge, Work life balance, leadership, Power, and Recognition) was employed for data collection.

**Results:**

The Cronbach’s alpha was 0.714 for the overall instrument. Statistically significant differences amongst the four generations of nurses emerged in the Work Value and Attitude scale in the construct of non-compliance (p = 0.007), technology challenge (p = 0.027), work-life balance (p < 0.001), and recognition (p < 0.001). No statistically significant differences were noted for the rest of the constructs.

**Discussion and conclusion:**

The findings of this study highlight that differences in work values and attitudes exist among nurses of different generations. Generation X are less likely to challenge the conventional norm and supervisors. Generation Y and Z are the most tech-savvy generations and can adapt quickly to new technology. There is also a greater emphasis on work-life balance as the generation gets younger. Generation Y and Z nurses perceived that younger nurses do not get due respect and recognition from their colleagues. Acknowledging the generational differences in work values and attitudes can facilitate nursing management to tailor strategies to improve individual and organisation performance while creating a work environment that enhances intergeneration harmony and teamwork.

**Supplementary Information:**

The online version contains supplementary material available at 10.1186/s12912-023-01256-2.

## Background and introduction

Among global scholars and practitioners, diminishing levels of employee work engagement have been one of the most alarming global economic phenomena which engender declining work performance and increasing employee turnover rates [1]. Work engagement, often defined as a positive, affective-motivational state of mind that is characterized by high levels of vigour, dedication and a strong focus on work [2], has been shown to coincide with high levels of individual and organizational performance standards, client satisfaction, job satisfaction, and employee retention [3, 4]. In the nursing context, further analysis also revealed that outcomes of nurses’ work engagement were decreased hospital mortality rates and significantly higher financial profitability of organizations [5]. With the continuous expansion of global competition, work engagement is a crucial driver of organisational success. Hence, researchers and human resource experts are increasingly focused on work engagement as a strategy to enhance the overall functionality of the organization, as well as the morale and commitment of individuals to leverage the organisational competitive edge [6].

A generation cohort is described as a group of individuals who share certain life stages and experiences during the same historic time frame [7]. The present nursing workforce comprises four generational cohorts that possess unique values and traits. The nursing profession is at a generational crossroad, experiencing a rapidly growing segment of the nursing workforce predominated by Generation Y/Millennials born between the years 1994 and 1980, and Generation Z born between years 1995 to 2015 [8, 9]. Existing generations of nurses include Baby Boomers born between the years 1944 to 1964, and Generation X, born between 1965 and 1979. There are prevalent beliefs about the existence of generation differences in work engagement in the workplace. Although research has shown that differences between work engagement and meaningful work amongst generational cohorts exist, results are still inconclusive as much research conducted was focused on the retiring cohort of Baby Boomers and Generation X [10]. Hence, further evidence is required to identify generational differences in work values, engagement, and satisfaction among the present nursing workforce.

Individuals in a generation cohort are born and share similar experiences of some major external events that attach and shape the individual’s values and attitudes. These emerged values have a significant effect on their lifestyles and remain relatively permanent throughout their lives [11]. As a result, the Generation Cohort theory espouses the homogeneity of this group of individuals as similar historic and social happenings create cohesiveness, influence, and underpin much of daily attitudes, engagement, and long-term behaviours [12]. With unique values, beliefs, and lifestyles which distinguish one generational cohort from another, such generational diversity will certainly add complexity to the workforce and challenges to providing optimal care delivery in the healthcare setting [13]. However, nursing leaders can view these differences in values and behaviours as potential strengths. With a better understanding of generationally driven variables, these insights can be leveraged to develop effective strategies to maintain the diverse yet shrinking nursing workforce. Generational differences can also be capitalized on and employed to create positive work environments as well as enhance quality and productivity to foster better patient care delivery. As generational differences become significantly more crucial as a diversity factor, a better understanding of the dynamics between work engagement and meaningful work across different generational cohorts is imperative to design the right approach for each organisation’s distinct parameters [14]. Therefore, this study aims to explore the differences in work values, attitudes, and engagement of different generational frontline nurses. The research question being addressed in this study was: What are the differences in work values, attitudes, and engagement among different generational frontline nurses?

## Research methodology

### Sampling

Data for this study were collected from full-time employed frontline nurses aged 21 years and above, including both registered and assistant nurses of a 795-bed general and acute care hospital in Singapore – Khoo Teck Puat Hospital. A power analysis was performed which determined a minimum sample requirement of n = 769 nurses with parameters set to achieve a statistical significance level of 0.05 and a power level of 0.80. To reduce sample bias considering that studies have revealed a significant impact of employment type and job designation on work values and attitudes [15, 16], this study aims to obtain a representative sample reflecting the proportion of job designations (5% Nursing supervisors, 79% Registered nurses, and 16% Assistant nurses) as well as the proportion of generation cohorts (0.4% Baby boomers, 7.2% Generation X, 68.4% Generation Y, and 24% Generation Z). The census-based estimates of the proportion of job designation and generation cohorts were obtained from the nursing administration department of the Hospital. Quota sampling was performed whereby participants were recruited until the quota of the pre-determined proportion of nurses was obtained.

### Measurement

Data was collected using a two-section survey. The first section collected unidentifiable demographic characteristics of the participants including age group (generation cohort), gender, ethnicity, nationality, religion, marital status, children, designation, educational qualification, and years of working experience. The second section was a cross-sectional, self-administered survey adapted from the study by Gursoy, Chi, and Karadag [17]. The Work Value and Attitude Scale comprises 25 items reflecting seven constructs of work attitude, values and engagement - Work Centrality (five items), Non-compliance (four items), Technology Challenge (three items), Work-life Balance (five items), Leadership (two items), Power (four items), and Recognition (two items). Each of the 25 subscales within the instrument uses a 5-point Likert Scale, ranging from 1 to 5, with score 1 representing strongly disagree, score 2 disagree, score 3 neutral, score 4 agree, and score 5 strongly agree.

### Data collection

Data was collected over five months from August 2020 to January 2021. Considering the restrictions to physical contact due to the COVID-19 pandemic and the varying shifts of frontline nurses, the survey was transcribed and administered through an online platform - form.gov.sg. In accordance with procedures approved by the ethics review board, an electronic mail was sent to all nursing supervisors within the institution to disseminate the study information to frontline nurses in all inpatient wards, accompanied by a participant information sheet and study recruitment poster which embedded a QR code for participants to assess the online questionnaire at convenience.

### Data analysis

Quantitative data were analysed through SPSS, using descriptive statistics to analyse the demographics of the sample and multiple tests to obtain inferential statistical data. A Cronbach Alpha’s reliability test was conducted to measure the reliability of each of the seven constructs in the Work Value and Attitude Scale. A Cronbach Alpha value of 0.60 to 0.70 indicates an acceptable level of reliability and 0.80 or greater is an excellent level [18]. The Shpiro-Wilks test for normality was performed to assert the symmetry of the sample means. As the result of the normality assumption test also indicated that the data are not normally distributed with a p-value of < 0.001 for all dimensions, non-parametric statistical tests were employed for further analysis of the data collected. Kruskal-Wallis statistical tests were then conducted to establish if there were any significant differences among different generation cohorts in each construct of the work value and attitude scale, and further elucidate if other demographical factors significantly impact nurses’ work values and attitudes. For binary variables such as gender and children, the Mann-Whitney U test was employed to identify differences between groups. To identify significant differences, Dunn’s post hoc test was performed for categorical variables that were significant which tested all possible 2-way comparisons of the medians between subgroups. Although significant differences between gender and children groups were noted in several constructs, no post hoc tests are available for binary variables.

### Ethical considerations

Permission to conduct the study was obtained from the chief nurse of the hospital, and ethical approval was obtained from the National Healthcare Group Domain Specific Review Board (DSRB). The participant information sheet described the purpose of the study, and provided the contact information of the principal investigator should respondents have any additional questions. The administered online survey was anonymous as the data collected involved no data attributable to personnel identification. As explicitly indicated in the participant information sheet, completion of the online survey indicated the participants’ implied consent to participate in the study. All data collected through the online platform was encrypted end-to-end and stored in an encrypted format, ensuring that only researchers can access and view responses with a private digital key. Upon completion of data collection, all data were kept in a hospital password-protected computer.

## Results

Of the 1364 full-time nurses employed within all inpatient wards, 778 (57%) responses were obtained at baseline. Although the obtained sample met the minimum requirement of 769 nurses to achieve statistical power, among the 778 participants, three (0.38%) participants had missing data and were hence excluded from further analysis. Table 1 presents the demographic profile of the analysed sample (n = 775). As presented in Table 1, the Generation Y cohort (Aged 26 to 40 years) accounted for 68.8% of the respondents. The majority of the respondents were females (92%), of Chinese (44.1%) ethnicity, Singaporean (38.6%), attained a Bachelor’s degree in Nursing (60.6%), and had 6 to 10 years of working experience (35.7%). While a diverse number of religions were reported among the respondents, the majority were Catholics (34.5%) or Christians (24.5%). Additionally, although 70.5% of the respondents had reported not having children, only 59.1% of the respondents were reported to be single. The obtained sample was also relatively proportionate to the projected population of frontline nurses in terms of the designations and four generation cohorts in the hospital (Tables 2 and 3).

**Table 1:** Demographic profile of respondents

Variable		Frequency (n)	Median	Percentage (%)
Age	Baby Boomers: 56 to 76 Years	36	80	4.6
	Generation X: 41 to 55 Years	28	79	3.6
	Generation Y: 26 to 40 Years	533	82	68.8
	Generation Z: 21 to 25 Years	178	85	23.0
Gender	Male	62	85.5	8
	Female	713	82	92
Ethnicity	Chinese	342	83	44.1
	Malay	111	84	14.3
	Indian	85	81	11.0
	Filipino	220	82	28.4
	Others	17	87	2.2
Nationality	Singapore	299	84	38.6
	Malaysia	163	83	21.0
	China	48	78	6.2
	Philippines	221	82	28.5
	Others	44	80.5	5.7
Religion	Buddhist	149	82	19.2
	Islam	116	84	15.0
	Hindu	49	81	6.3
	Christian	190	82.5	24.5
	Catholic	267	82	34.5
	Others	4	82	0.5
Marital Status	Single	458	83	59.1
	Married	307	81	39.6
	Divorced/Separated	9	88	1.2
	Widowed	1	87	0.1
Children	Yes	229	81	29.5
	No	546	83	70.5
Designation	Enrolled Nurse	125	82	16.1
	Staff Nurse	604	83	78
	Nursing Officer	46	81	5.9
Educational Qualification	Certificate in Nursing	50	83.5	6.5
	Diploma in Nursing	235	83	30.3
	Bachelor’s Degree	470	82	60.6
	Master’s Degree	17	82	2.2
	Others	3	76	0.4
Years of Experience	< 2 Years	145	85	18.7
	2 to 5 Years	218	83	28.1
	6 to 10 Years	277	83	35.7
	11 to 20 Years	103	80	13.3
	> 20 Years	32	80	4.1

**Table 2:** Projected and sample proportion of job designation

Designation	Projected proportion	Sample
Supervisors	5%	5.9%
Staff Nurses	79%	78%
Assistant Nurses	16%	16.1%

**Table 3:** Projected and sample proportion of generation cohort

Generation Cohort	Projected proportion	Sample
Baby Boomers	4%	4.6%
Generation X	7.2%	3.6%
Generation Y	68.4%	68.8%
Generation Z	24%	23%

The Cronbach’s alpha tests established adequate to excellent reliability of all seven constructs, with Cronbach’s alpha scores between 0.70 and 0.817, except for the “work centrality construct” (0.663) and “technology challenge construct” (0.604). Results of the Kruskal-Wallis test presented in Table 4 revealed statistically significant differences among generational cohorts in four constructs – non-compliance, technology challenge, work-life balance, and recognition. The generational effects of each of the seven work value and attitude constructs are discussed below.

**Table 4:** Median score of generational cohorts for the seven work values and attitude constructs

	Baby Boomers	Generation X	Generation Y	Generation Z	p-value
**Work Centrality**					
Median	19	19	19	19	0.951
**Non-Compliance**					
Median	12	11	12	12	**0.007**
**Technology Challenge**					
Median	7	7	6	8	**0.027**
**Work-Life Balance**					
Median	16	17	18	21	**< 0.001**
**Leadership**					
Median	8	8	8	8	0.564
**Power**					
Median	12	12	12	13	0.117
**Recognition**					
Median	4.5	4	5	6	**< 0.001**

### Work centrality

The first factor “work centrality” refers to an individual’s perception of the job’s importance and job orientation which encompasses job security, idealism, professional development, and promotion. Primary analysis revealed no significant differences between the generational cohorts on work centrality (p = 0.951).

### Non-compliance

“Non-compliance” deals with extrinsic behaviour including the need to challenge conventional workplace norms, rules, and management. Significant generational differences were found in the “non-compliance” construct (p = 0.007). Median scores for the construct of non-compliance were similar across all generational groups (median = 12) except for Generation X – age 41 to 55 years (median = 11). To further elucidate significant differences between the generational groups, results of the post hoc test showed significant differences in the median scores for the “non-compliance” construct between Generation Y and Generation Z (p = 0.042), Generation Y and Baby boomers (p = 0.024), as well as between Generation X and Baby boomers (p = 0.019). With the lowest non-compliance score, these results suggest Generation X is most compliant with rules and regulations as compared to the younger generations.

### Technology challenge

“Technology challenge” refers to the impact of technology on individuals’’ work. Significant generational differences in technology challenge were also reported (p = 0.027). Median scores for the “technology challenge” construct were similar among the older generations (median = 7), while Generation Y and Generation Z revealed lower median scores (median = 6). These results suggest that Generation Y and Generation Z are the most tech-savvy generations and can adapt quickly to new technology as Generation Y were the first wave of the digital generation born into the world of technology while Generation Z was born into a highly developed digital era.

### Work-life balance

“Work-life balance” emphasizes the need for a distinct separation of work and personal life, including priories and importance of work. Significant differences in work-life balance were reported among the different generational cohorts (p < 0.001) which post hoc test revealed significantly different median scores for the “work-life balance” construct between all generational cohorts (p < 0.001). The results revealed that there is a greater emphasis on work-life balance as the generation cohort gets younger as evidenced by the increasing median work-life balance scores from Baby Boomers to Generation Z. This suggests that younger generations possess high expectations for a healthy work-life balance as they value flexibility and freedom in their workplaces while the older generations hold greater emphasis on discipline and hard work.

### Leadership

“Leadership” focuses on the individual’s need for leadership and direction at work. No significant differences among the generational cohorts were reported on leadership (p = 0.564). Median scores between all groups were consistent (median = 8).

### Power

“Power” refers to the tendency of individuals striving for power, control, and to be in command of their tasks as well as others. No significant differences were found between generational cohorts for the construct “power” (p = 0.117). All groups also reported similar median scores (median = 12) except Generation Z (Median = 13).

### Recognition

Lastly, “recognition” emphasizes the perception of individuals in the younger generation. This factor suggests that younger nurses are not respected or treated like a kid due to their age. There were significant differences among generations for recognition (p < 0.001). Median scores for the construct “recognition” varied between all four generational cohorts whereby Generation Z had the highest median score (median = 6) while Generation X had the lowest median score (median = 4). Post hoc tests revealed significant differences in median scores between all generational cohorts and the Baby Boomer generation. In addition, significant differences in median scores were also identified between Generation X and Generation Z (p = 0.033). These results suggest that Generation Y and Generation Z nurses perceive that younger nurses do not get their due respect and recognition (p < 0.001).

## Discussion

This study aims to explore the differences in work values, attitudes, and engagement of different generational frontline nurses. With the in-depth knowledge of intergenerational work value, attitude, and engagement differences, this study seeks to provide strategies to improve work environments and conditions to encourage intergenerational harmony and teamwork among nurses.

The findings of this study revealed generational differences in the constructs of non-compliance, work-life balance, and recognition relating to nurses’ work values, engagement, and satisfaction. Compared to the other 3 generational cohorts, Generation X had lower median non-compliance scores which suggests a lesser need to challenge workplace norms such as dress code, flex time, employee-supervisor relations, cliche rules, and management. This finding is deemed to have significant implications on managerial styles including workplace flexibility relating to work hours, shifts, and incentives. The result is supported by Stevanin et al. [19] which indicated that Generational X had the lowest scores with regards to flexibility while Generation Y had the highest score also revealed in the present study whereby post-hoc test results revealed significant differences in non-compliance scores of the Generation Y cohort with Generation Z and Baby Boomers. In contrast to the other generations, the Generation Y cohort values flexibility and autonomy, is adaptable to change, participates in discovering new knowledge, and challenges their own and other’s assumptions [20] which may be seen as a generational-specific attitude which entails more considerations by nursing leaders in the workplace. The lack of due consideration may result in tension, friction, and conflict among employees thereby decreasing job satisfaction and productivity. Managers need to understand individuals from different generational cohorts possess varying value sets and hence synergize the strengths of the cohorts to successfully engage intergenerational groups for workplace harmony. For instance, organisational flexibility can be leveraged to optimize performance and productivity, especially in healthcare environments with resource restraints whereby flexible work practices can effectively and efficiently cover staffing needs such as short-term leaves of absence [21]. The flexibility of work practices can be conserved as a diversity initiative to retain a more diverse workforce which caters to the generational diversity, needs, and priorities of nurses.

Additionally, the results of this study suggest that the older generations including the Baby Boomers and Generation X presented higher technology challenge scores as compared to the younger Generation Y and Generation Z. Findings are consistent with existing literature that Generation Y and Z are quick to adapt to new technology as they are born into the age of technology, virtual space, and social media [22]. This can be employed as a strength in the organization whereby technology is embraced through skills upgrading and technological advancements. Tailored training may be provided to the pioneering generations such as Generation X and Baby Boomers to upgrade their skills and knowledge in the use of technology, while younger generations including the Generation Y and Generation Z nurses who are highly qualified and adaptable to the use of new technology may be empowered as champions in healthcare technological advancements to improve and streamline care processes with the use of new technology.

Results of the present study further revealed a statistically significant difference and positive correlation between the younger generations and the need for work-life balance. While baby boomers presented with the lowest scores for the work-life balance construct, Generation Z presented with the highest scores in addition to the power construct. This requires significant consideration in the leadership and management of the younger generation nurses as it reveals that despite the need for separation of work and personal life adopted by the younger generation, there is nonetheless a strive for power, authority, and organization of their tasks and duties. Nevertheless, it is essential to consider the demographical factors which influence the work value and attitudes of individuals including supervisory and upper-level managerial positions. Considering that the majority of the supervisory and managerial positions are attained by baby boomers and the Generation X cohort with substantial working experience in the organization or speciality, it may be challenging for them to understand the work value and attitudes of the younger generation of nurses hold greater value to their personal life. Hence, consideration of what is most valued and prioritised by different generational cohorts may be an effective strategy for administrators in the approach of management and retention of the younger generation cohort while maintaining work satisfaction. Sherman, Saifman, Schwartz, and Schwartz [23] further support that although younger generational cohort nurses such as the millennials do recognize the importance of nursing leadership in the delivery of patient care, work-life balance is a significant deterrent to the role. As Singapore and the international healthcare system faces a shrinking nursing workforce, it is crucial to not only retain but also empower the younger generations which form the largest generation within nursing to find their place in nursing leadership.

However, findings have also elucidated concerns towards support and respect towards the younger generation of the nursing workforce. Generation Y and Z which represents the youngest of the nursing generational cohort had presented with the highest recognition scores which suggested feelings that individuals do not get the due respect or consideration because of their age. Hence, intergenerational collaboration is critical that not only are the younger generational cohorts be willing to take on shared responsibility to shape the future of the profession through power and leadership, autonomy through the sense of independence and opportunities, as well as guidance such as through mentorship to bridge the knowledge and experience gap from the older generations are also essential.

## Limitations

Just as with any other study, this study has limitations. As data of this study are gathered using a self-administered online survey through convenience sampling, results may not be the most representative of the studied hospital although the sample is relatively proportionate to the projected population of frontline nurses in the studied hospital, reflecting the proportion of the four generation cohorts and designations in the hospital. Additionally, as the study was only conducted in one local governmental hospital, findings may not be generalized to other healthcare organizations such as private hospitals due to differing operational policies and human resource management. Future studies should consider a larger scale sample involving multiple healthcare organizations and may also seek to study other factors that may impact the work attitude and values of nurses including different specialization roles such as the intensive care and emergency department. Moreover, considering that this study had elucidated multiple demographical variables such as gender, ethnicity, working experience, and educational qualifications which have reported significant differences in the various work attitude and values, future studies may further examine the socio-demographical impact on work attitudes and values among the different generations.

## Conclusion

In conclusion, the results of this study elucidate that there are significant multigenerational differences in work attitudes and values among frontline nurses. Knowledge and understanding of these generational differences and similarities are crucial and should be actively considered by administrators or managers to foster a harmonious inter-generational working environment while leveraging on these values and attitudes to increase productivity while retaining staff. Findings suggest that the younger generation including millennials and generation Z work to live with greater emphasis on work-life balance, while the older generational cohorts including baby boomers live to work with a greater commitment to their jobs. The younger generation cohorts also desire greater power and autonomy at work with recognition and respect despite their young age. Identification of these generational differences and issues will also allow nursing leaders to better strategize approaches such as workplace flexibilities relating to shifts, working hours, and incentives to better cater to the needs of the different generational cohorts while increasing employee work satisfaction, morale, and care delivery to patients.

## Electronic supplementary material

Below is the link to the electronic supplementary material.

Supplementary Material 1

## Data Availability

The datasets used and/or analysed during the current study are available from the corresponding author on reasonable request.

## References

[1] Motyk B (2018). Employee engagement and performance: a systematic literature review. Int J Econ Manag.

[2] Bakker AB, Albrecht S (2018). Work engagement: current trends. Career Dev Int.

[3] Tabasum S, Shikh AR (2022). Impact of employee engagement on employee performance with mediating effect of employee retention. Int J Soc Sci Entrep.

[4] Yang X, Feng Y, Meng Y, Qiu Y (2019). Career adaptability, work engagement, and employee well-being among chinese employees: the role of guanxi. Front Psychol.

[5] Sayed SS, Ahmed MZ, Bakr MM, Sherief NM (2019). Work engagement in nursing: a concept analysis. Menoufia Nurs J.

[6] Hamadamin HH, Atan T (2019). The impact of strategic human resource management practices on competitive advantage sustainability: the mediation of human capital development and employee commitment. Sustainability.

[7] Rudolph CW, Rauvola RS, Costanza DP, Zacher H (2021). Generations and generational differences: debunking myths in organizational science and practice and paving new paths forward. J Bus Psychol.

[8] Waltz LA, Muñoz L, Johnson HW, Rodriguez T (2020). Exploring job satisfaction and workplace engagement in millennial nurses. J Nurs Manag.

[9] Chicca J (2019). A new generation of nurses is here: strategies for working with Generation Z. Am Nurse Today.

[10] Ludwig CM, Geisler AN, Fernandez JM, Battaglia G, Andorfer C, Hinshaw MA (2020). The challenge of change: resilience traits in women’s dermatological Society Forum participants by generation. Int J Womens Dermatol.

[11] Ting H, Lim T, de Run EC, Koh H, Sahdan M (2018). Are we Baby Boomers, Gen X and Gen Y? A qualitative inquiry into generation cohorts in Malaysia. Kasetsart J Soc Sci.

[12] Christensen SS, Wilson BL, Edelman LS (2018). Can I relate? A review and guide for nurse managers in leading generations. J Nurs Manag.

[13] Douglas K, Gray S (2020). Generational complexities present new challenges for nurse leaders. Nurse Lead.

[14] Arslan A, Ahokangas P, Haapanen L, Golgeci I, Tarba SY, Bazel-Shoham O (2020). Generational differences in organizational leaders: an interpretive phenomenological analysis of work meaningfulness in the nordic high-tech organizations. Technol Forecast Soc Change.

[15] Hegney D, Plank A, Parker V (2006). Extrinsic and intrinsic work values: their impact on job satisfaction in nursing. J Nurs Manag.

[16] Johnson D, Lake CJ (2019). Contingent worker monetary influence, work attitudes and behavior. Pers Rev.

[17] Gursoy D, Chi CG, Karadag E (2013). Generational differences in work values and attitudes among frontline and service contact employees. Int J Hosp Manag.

[18] Ursachi G, Horodnic IA, Zait A (2015). How reliable are measurement scales? External factors with indirect influence on reliability estimators. Procedia Econom Financ.

[19] Stevanin S, Palese A, Bressan V, Vehviläinen-Julkunen K, Kvist T (2018). Workplace-related generational characteristics of nurses: a mixed-method systematic review. J Adv Nurs.

[20] Jung H, Yoon H (2021). Generational effects of workplace flexibility on work engagement, satisfaction, and commitment in south korean deluxe hotels. Sustainability.

[21] MacPhee M, Borra LS (2013). Flexible work practices in nursing.

[22] Andrea B, Gabriella H, Timea J (2016). Y and Z generations at workplaces. J Compt.

[23] Sherman RO, Saifman H, Schwartz RC, Schwartz CL (2015). Factors that lead Generation Y nurses to consider or reject nurse leader roles. NursPlus Open.

