# Supplementary material for: Generational effect on nurses’ work values, engagement, and satisfaction in an acute hospital
Source: BMC Nurs. 2023 Mar 30;22:88. doi: 10.1186/s12912-023-01256-2 (PMC10061355; doi:10.1186/s12912-023-01256-2)
Supplement: Supplementary file 1 — Supplementary Material 1 [file 12912_2023_1256_MOESM1_ESM.docx]

**Work Values and Engagement Questionnaire**

Age:  21-25 years  26-40 years  41-55 years  56-76 years

Gender:  Male  Female

Ethnicity:  Chinese  Malay  Indian  Others ________­­­_________

Nationality:  Singapore  Malaysia  China  Philippines  Myanmar  Others _____________

Religion:  Christian  Catholic  Buddhist  Taoist  Islam  Hindu  Others _____________

Marital Status:  Single  Married  Divorced/ Separated  Widowed  Others _______________

Children:  Yes  No

Designation:  SN/SSN  EN/PEN  ANC/NC/NM/NE/SNC/SNM

Educational Qualification:  Certificate in Nursing (ITE)  Diploma in Nursing

 Advanced Diploma in Nursing  Bachelor’s Degree in Nursing

 Master’s Degree  Doctorate in Nursing/ PhD

 Others ____________________________________________

Years of working experience:  <2 years  2-5 years  6-10 years  11-20 years  >20 years

In answering the following questions, it is extremely important that you refer to your own generation, sincerely express your opinion and answer according to what you actually do in everyday work life.

| Factors | Strongly Disagree | Disagree | Neutral | Agree | Strongly Agree |
| --- | --- | --- | --- | --- | --- |
| **Work Centrality** | | | | | |
| Job security is very important for me | 1 | 2 | 3 | 4 | 5 |
| I am willing to work hard and long hours | 1 | 2 | 3 | 4 | 5 |
| When it comes to my job, I am very idealistic and driven | 1 | 2 | 3 | 4 | 5 |
| I take my job and professional development very seriously | 1 | 2 | 3 | 4 | 5 |
| I am willing to wait for my turn for promotions and rewards | 1 | 2 | 3 | 4 | 5 |
| **Non-Compliance** | | | | | |
| I am likely to challenge workplace norms such as dress codes,  flex time, and employee-supervisor relations | 1 | 2 | 3 | 4 | 5 |
| I truly believe the cliché that rules were meant to be broken | 1 | 2 | 3 | 4 | 5 |
| I have low tolerance for bureaucracy and rules | 1 | 2 | 3 | 4 | 5 |
| I am deeply cynical about management | 1 | 2 | 3 | 4 | 5 |
| **Technology Challenge** | | | | | |
| Technology makes my job harder | 1 | 2 | 3 | 4 | 5 |
| I feel like my computer will replace me | 1 | 2 | 3 | 4 | 5 |
| Using latest technology makes my job easier | 1 | 2 | 3 | 4 | 5 |
| **Work-Life Balance** | | | | | |
| I work to live, not the other way around | 1 | 2 | 3 | 4 | 5 |
| My philosophy is “Leave work at work.” | 1 | 2 | 3 | 4 | 5 |
| I will not sacrifice my leisure time for the company | 1 | 2 | 3 | 4 | 5 |
| My priorities are with my friends and my family, not the boss | 1 | 2 | 3 | 4 | 5 |
| I want to work as many hours as I have to but not a minute longer | 1 | 2 | 3 | 4 | 5 |
| **Leadership** | | | | | |
| I work best when there is strong leadership | 1 | 2 | 3 | 4 | 5 |
| I work best when there is direction | 1 | 2 | 3 | 4 | 5 |
| **Power** | | | | | |
| I strive to be “in command” when I am working in a group | 1 | 2 | 3 | 4 | 5 |
| I strive to gain more control over the events around me at work | 1 | 2 | 3 | 4 | 5 |
| I find myself organizing and directing the activities of others | 1 | 2 | 3 | 4 | 5 |
| I take moderate risks and stick my neck out to get ahead at work | 1 | 2 | 3 | 4 | 5 |
| **Recognition** | | | | | |
| They treat younger employees like kids | 1 | 2 | 3 | 4 | 5 |
| No one respects younger employees because they are young | 1 | 2 | 3 | 4 | 5 |

Adapted from “Generational Differences in Work Values and Attitudes Among Frontline and Service Contact Employees” by D. Gursoy, C. G. Chi and E. Karadag, 2013, International Journal of Hospitality Management, 32, 40-48. Copyright 2013 by Elsevier Ltd.
